# Alterations in Regional Brain Regional Volume Associated with Dioxin Exposure in Men Living in the Most Dioxin-Contaminated Area in Vietnam: Magnetic Resonance Imaging (MRI) Analysis Using Voxel-Based Morphometry (VBM)

**DOI:** 10.3390/toxics9120353

**Published:** 2021-12-15

**Authors:** Hoa Thi Vu, Thao Ngoc Pham, Takashi Yokawa, Muneko Nishijo, Tai Pham The, Quyet Do, Yoshikazu Nishino, Hisao Nishijo

**Affiliations:** 1 Department of Public Health, Kanazawa Medical University, Uchinada 920-0293, Japan; vuhoa5593hvqy@gmail.com (H.T.V.); ni-koei@kanazawa-med.ac.jp (M.N.); ynishino@kanazawa-med.ac.jp (Y.N.); 2 Biomedical and Pharmaceutical Research Center, Vietnamese Military Medical University, Hanoi 10000, Vietnam; phamngocthaovmmu@gmail.com (T.N.P.); taithuy@kanazawa-med.ac.jp (T.P.T.); dobaquyet@yahoo.com (Q.D.); 3 Kobe BMA Laboratory, BioView Inc., Kobe 650-0047, Japan; yokawa@bioview.co.jp; 4 Faculty of Medicine, University of Toyama, Toyama 930-0194, Japan

**Keywords:** dioxin, neuro imaging analysis, brain regional volume, adults, Vietnam

## Abstract

To clarify the influence of dioxin exposure on brain morphometry, the present study investigated associations between dioxin exposure at high levels and brain structural irregularities in 32 Vietnamese men. Two exposure markers were used: blood dioxin levels, as a marker of exposure in adulthood, and perinatal dioxin exposure, estimated by maternal residency in a dioxin-contaminated area during pregnancy. All subjects underwent brain magnetic resonance imaging (MRI) scans. We analyzed correlations between regional gray matter volumes and blood dioxin levels, and compared regional volumes between men with and without perinatal dioxin exposure using the voxel-based morphometry (VBM) tool from Statistical Parametric Mapping 12 (SPM12). Blood 2,3,7,8-tetrachlorodibenzo-p-dioxin (TCDD) was associated with low volume of the medial temporal pole and fusiform gyrus. Toxic equivalency (TEQ)-PCDDs were correlated with low medial temporal pole volume. However, 1,2,3,4,7,8-HxCDD was associated with high middle frontal gyrus and cerebellum volume. In men with perinatal dioxin exposure, the left inferior frontal gyrus pars orbitalis volume was significantly lower than in those without perinatal exposure. These results suggest that dioxin exposure during the perinatal period and in adulthood may alter regional brain volume, which might lead to cognitive deficits and unusual social emotional behavior in Vietnamese men living in dioxin-contaminated areas.

## 1. Introduction

During “Operation Ranch Hand” between 1961 and 1971, the US Armed Forces sprayed large quantities of herbicides, such as Agent Orange, causing widespread dioxin contamination in Southern Vietnam. Nearly 50 years later, Dwernychuk (2005) reported that dioxin residues remained in both the environment and in humans residing in the sprayed areas of Vietnam, particularly around several former US airbases located in Bien Hoa, Da Nang, and Phu Cat [1]. Bien Hoa airbase is recognized as the largest hotspot of dioxin contamination in Vietnam; the Office of the Vietnam National Steering Committee 33 and Hatfield Consultants (2011) reported the detection of large quantities of 2,3,7,8-tetrachlorodibenzo-p-dioxin (TCDD), as high as 61,400 pg/g dry weight in soil, originating from Agent Orange, in samples from the Bien Hoa airbase [2]. Blood dioxin concentrations in men living around the Phu Cat and Bien Hoa airbases were four to five times higher than those in an unsprayed area in Northern Vietnam [3]. TCDD concentrations in blood samples of military workers in Bien Hoa airbase were two to five times higher than those in people working in Da Nang and Phu Cat airbases, respectively [4].

Our previous Vietnam-based study reported that perinatal exposure to toxic equivalency values of polychlorodibenzodioxins/furans (TEQ-PCDD/Fs) was associated with poor neurodevelopmental scores for all domains, and that TCDD exposure was associated with increased autistic traits in 3-year-old children in an area around Da Nang airbase [5]. In the areas around Bien Hoa airbase, where TCDD levels in breast milk were two times higher, but TEQ-PCDD/Fs levels were two-thirds of those in Da Nang; we found that boys with high exposure to TCDD and other PCDD congeners showed lower expressive and composite language scores and lower gross motor scores than those in a low exposure group [6]. In 2015, neonatal electroencephalography (EEG) was measured in a group of neonates recruited in the same area, and perinatal dioxin exposure was found to be associated with altered EEG power and coherence, poor neurodevelopment, and poor gaze behavior at 2 years of age [7,8]. These findings suggest that dioxin exposure may influence development of the fetal brain, leading to neurodevelopment disorders such as autism spectrum disorder (ASD) in later life.

ASD is a neurodevelopmental disorder that presents with social communication deficits and restricted repetitive patterns of behavior, interests, and activities. Voxel-based morphometry (VBM) is a technique applied to brain MRI to investigate focal differences in brain anatomy. Several VBM studies have demonstrated structural brain irregularities in patients with ASD [9,10,11]. These studies raised the possibility that children with autistic traits caused by dioxin exposure might have different brain structures to children not exposed to dioxin. However, the MRI examination of children is difficult because of the requirement for sedation. Therefore, in the present study, we recruited the fathers of children in our birth cohort recruited in Bien Hoa in 2015, and used VBM to investigate associations between dioxin exposure and brain structural irregularities in men living near Bien Hoa airbase.

## 2. Materials and Methods

### 2.1. Study Subjects

In 2018, 55 fathers of children living in communes around Bien Hoa airbase whose neonatal EEGs were examined in 2015 were invited to join a survey to investigate their dioxin exposure levels according to blood analysis, and 40 of them responded (73% participant rates). In 2019, these 40 men whose blood dioxin levels were measured were invited to brain MRI examinations, but only 33 men (60%) participated in the present study. Seven men were too busy with their works or families to join the study on the examination days. Moreover, one participant was considered an outlier because of a very high TCDD blood level (371.5 pg/g lipid) and was excluded from the data analysis. Consequently, 32 men were included in the final data analysis of the present study. 

As regional brain volumes in adulthood may be programmed during the fetal period [12], we investigated associations between the men’s regional brain volumes and perinatal dioxin exposure estimated from their mothers’ residential information before their births. The subjects were interviewed to retrieve details of their mothers’ residency before they were born, and 12 mothers (37.5%) were found to have lived in Bien Hoa during pregnancy (1970 to 1992) with one of our subjects, suggesting that these men might have been exposed to a high level of TCDD during the fetal period. Schecter et al. (2001) reported very high levels of TCDD in breast milk; 133–1832 (pg/g lipid) in samples collected in 1970–1973 and 2.1–11 (pg/g lipid) in 1985–1988 among Bien Hoa residents, although the levels were decreased over years [13]. They also reported high blood levels of TCDD, 2.0–164 (pg/g lipid) in samples collected in 1999 [13]. Furthermore, it is supposed that all mothers fed babies breast milk before weaning, since infant formula was not readily available and not a common feeding method for residents in Bien Hoa after the long wartime. These findings suggest that the mothers of our subjects as well as the subjects, who had lived in Bien Hoa before and after birth, were exposed to TCDD originating from Agent Orange even after spraying herbicide was terminated.

Written informed consent was obtained from all men according to a process reviewed and approved by the Health Departments of Bien Hoa City and Dong Nai Prefecture. The institutional ethics board for medical and health research involving human subjects at Kanazawa Medical University approved the study design (Approval Code: No. I-424, Approval Date: 19 September 2017).

### 2.2. Dioxin Measurements in Whole Blood

Around twenty milliliters of venous blood were collected by nursing staff at Bien Hoa health center. Whole blood samples were frozen and transferred to Kanazawa Medical University in Japan, and the levels of 17 2,3,7,8-substituted PCDD/Fs (7 congeners of PCDDs and 10 congeners of polychlorinated dibenzofurans; PCDFs) and four non-ortho-polychlorinated biphenyl (PCB) congeners (3,3,4,4-tetra-chlorinated biphenyl [TCB#77]; 3,4,4,5-tetra-chlorinated biphenyl [TCB#81]; 3,3,4,4,5-penta-chlorinated biphenyl [PeCB#126]; and 3,3,4,4,5,5-hexa-chlorinated biphenyl [HxCB#169]) were measured. Whole blood samples were frozen and dehydrated in an EYELA freeze dryer (FDU-1200; Tokyo-rika Inc., Tokyo, Japan), and fat was extracted using an ASE-200 accelerated solvent extractor (Dionex Corporation, Sunnyvale, CA, USA). Then, 13C-labeled 2,3,7,8-substituted PCDDs/Fs (DF-LCS-A40; Wellington Inc., Ontario, Canada) and 13C-labeled dioxin-like (dl)-PCBs (DLPCB-CL-A20; Kanto Chemical Co., Inc., Tokyo, Japan) were added to samples as internal standards. After chromatography using a multilayered silica gel column to purify samples, a single-layered column of activated carbon was used to separate and collect the PCDD/Fs and non-ortho PCBs fraction. The final extracted solution was concentrated by evaporation, and 17 PCDD/F congeners and 4 non-ortho PCB congeners were quantified using a gas chromatograph (HP-6980; Hewlett-Packard, Palo Alto, CA, USA) equipped with a high-resolution mass spectrometer (MStation-JMS700; JEOL, Tokyo, Japan). The limit of detection (LOD) for each congener of PCDDs/Fs/ non-ortho PCBs with numbers of samples lower than LODs were shown in Table 1. For quantification of each congener, measurement values were used if they were higher than LODs, but, if not (below LODs), halves of the LODs were used as a quantification values.

The TEQ values of PCDDs, PCDFs, and non-ortho PCBs in each sample were calculated by multiplying each congener concentration by its TEQ factor referenced from the World Health Organization 2005 TEQ factors list [14]. The geometrical means and ranges of 17 PCDD/F congeners, TCB#77, and HxCB#169, and the TEQs for PCDDs, PCDFs, PCDDs/Fs, non-ortho PCBs, and PCDDs/Fs/non-ortho PCBs in the blood after lipid base calculations are shown in Table 1. Because of levels below detection limits for almost all samples (>93%), the values of TCB#81 and PeCB#126 congeners are not shown in Table 1.

### 2.3. MRI Data Acquisition and Voxel-Based Morphometry

All subjects underwent brain MRI scans on a Siemens Magnetom Trio Tim system 3T-scanner (Siemens, Erlangen, Germany) at the Department of Diagnostic Imaging in Dong Nai General Hospital, Vietnam. High-resolution T1-weighted images with good contrast between gray and white matter were collected. The image parameters included: TR = 1520 ms, TE = 2.07 ms, flip angle = 9°, slice thickness = 0.9 mm, 192 slices, and field of view =230 × 230 mm.

All images subjected to voxel-based morphometry (VBM) analysis were preprocessed using the Computational Anatomy Toolbox (CAT12, vCAT12.7-RC1; Structural Brain Mapping group, Jena University Hospital, Jena, Germany; http://dbm.neuro.uni-jena.de/cat/ (accessed on 14 December 2021)) in the Statistical Parametric Mapping 12 software package (SPM12; The Wellcome Centre for Human Neuroimaging, London, UK; https://www.fil.ion.ucl.ac.uk/spm/software/spm12/ (accessed on 14 December 2021)) running under MATLAB software (The Mathworks, Inc., Natick, MA, USA). The image preprocessing steps performed were based on a standard protocol (http://www.neuro.uni-jena.de/cat12/CAT12-Manual.pdf (accessed on 14 December 2021)). All T1-weighted images were corrected for rough bias, affine registered to a template image in MNI152 space, then segmented into gray matter, white matter, and cerebrospinal fluid maps using the segmentation tools in SPM12 [15]. The segmented images were spatially normalized to the same total brain volume using the DARTEL algorithm [16] and smoothed with an isotropic Gaussian kernel (full-width half-maximum = 8 mm).

The total gray matter, white matter, cerebrospinal fluid, and intracranial volumes were estimated using CAT12. The total brain volume was calculated from the sum of the volume of gray and white matter [17].

### 2.4. Statistical Analysis

The correlations of VBM-derived regional brain volumes with blood dioxin levels were evaluated using a multiple regression model in SPM12 using the data of the 32 men. The normalization process of the DARTEL algorithm normalized the global brain volume of each subject to the same value while conserving regional differences in brain matter volume. Blood dioxin congener levels were treated as a covariate of interest after base-10 logarithm transformation to improve normality. The total intracranial brain volume and age of the subjects were treated as confounding covariates. Two linear contrasts (positive or negative correlations) were used to estimate associations between blood dioxin levels and brain regions.

Two-sample *t*-tests in SPM12 were used to compare regional brain volumes between subjects with and without perinatal dioxin exposure indicated by maternal residency in Bien Hoa during pregnancy. These tests were performed after adjusting for total intracranial brain volume and age.

The Montreal Neurological Institute (MNI) coordinates of the voxel of maximal statistical significance in each cluster (connected voxels defined by a prespecified statistical threshold) were extracted. For all VBM analyses, the cluster-based false-discovery rate (FDR) for multiple comparisons combined with the peak detection threshold was used for testing statistical significance [18]. The Anatomy toolbox in SPM12 [19] was used to identify anatomical regions showing significant results in the VBM analysis. *p* < 0.05 was considered statistically significant in all tests.

SPSS version 22.0 (IBM, Armonk, NY, USA) was used for statistical analyses of global brain volume. General linear models were used to compare global brain volume parameters (independent variables) between subjects with and without perinatal dioxin exposure (a fixed factor), after adjusting for confounding factors correlated with global brain volume (covariates), including age (years) and height (cm).

## 3. Results

### 3.1. Relevant Factors Associated with Brain Volume and Dioxin Exposure

The characteristics of the subjects are shown in Table 2. The mean (±standard deviation (SD)) age and educational years of the subjects were 35.5 ± 5.9 and 11.9 ± 3.1 years, respectively. Twenty-two men (68.8% of the total subjects) graduated high school and/or schools with upper levels. Twenty-six men (81.3%) consumed alcohol, but only four men (12.5%) took alcohol every day. Their mean body mass index (BMI) was 24.2 and 14 men (43.8%) were obese (BMI ≥ 25). Most subjects were right-handed (87.5%). The mean (±SD) residential period near to Bien Hoa airbase was 22.1 ± 14.6 years. Five men (15.6%) were mechanics who worked on machines that belonged to the airbase, and four men were soldiers. Ten men (31.3%) used herbicides and pesticides in the growing of vegetables in their gardens.

The dioxin and non-ortho PCB concentrations in the blood were compared according to occupation, consumption of food grown in the airbase, use of herbicides and pesticides, and length of residency. The concentrations of TCDD and TEQs of PCDDs and PCDDs/Fs/non-orthoPCBs were significantly higher in people who worked in the airbase than in those with other jobs (t = −3.27, *p* = 0.003; t = −3.41, *p* = 0.002; t = −3.28, *p* = 0.003, respectively), and in subjects using herbicides and pesticides than in those not using them (t = −2.41, *p* = 0.022; t = −2.69, *p* = 0.012; t = −2.29, *p* = 0.030, respectively). However, these differences were not statistically significant after adjusting confounding factors. The geometric mean TCDD concentration of two men who consumed dioxin-contaminated food grown in the airbase was four times higher than that of other subjects. There were no significant correlations between the concentrations of any of the PCDDs/Fs/non-orthoPCB congeners and the length of residency.

### 3.2. Associations between Current Dioxin Exposure (Indicated by Blood Dioxins) and Brain Volume

#### 3.2.1. Global Brain Volume Analysis

We analyzed simple correlations (Spearman’s ρ) between global brain volume and levels of 17 congeners of PCDD/Fs and 2 congeners of non-ortho-PCBs (data not shown). Global gray matter volume (cm^3^) was significantly inversely correlated with 1,2,3,4,7,8-Hexachlorodibenzo-p-dioxin (HxCDD) (ρ = −0.378, *p* = 0.033), but positively correlated with Octachlorodibenzo-p-dioxin (ρ = 0.369, *p* = 0.038) and 1,2,3,4,6,7,8-Heptachlorodibenzofuran (ρ = 0.354, *p* = 0.047). A significant positive correlation was also found between global cerebrospinal fluid volume (cm^3^) and HxCB#169 (ρ = 0.446, *p* = 0.011). However, these correlations between global gray matter and dioxin and PCB congeners were not significant after adjusting for confounding factors such as age and height (data not shown). No significant correlations with any dioxin congeners in the blood were found for total white matter volume, total brain volume, and total intracranial volume, even before adjusting for confounding factors.

#### 3.2.2. VBM Analyses; Brain Regions in Which Gray Matter Volume Correlated with Blood Dioxin Levels

**Figure 1:**
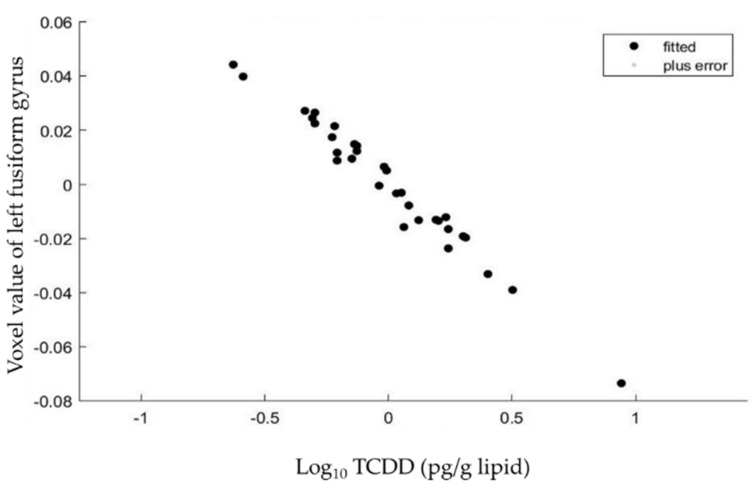
Relationships between the voxel values of the left fusiform gyrus at [−27 (x), 8 (y), −47 (z)] and blood TCDD levels in the 32 subjects. There was a significant and inverse correlation between the two parameters after adjusting for total intracranial volume and age of the subjects.

**Figure 2:**
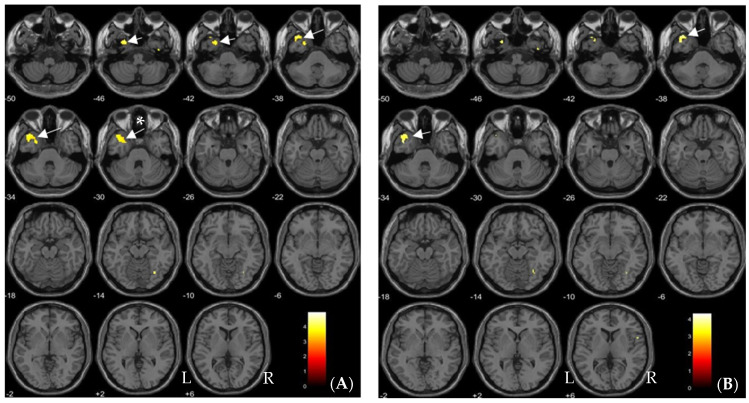
Brain regions showing significant negative correlations with blood levels of TCDD (**A**) and TEQ-PCDDs (**B**) (FDR-corrected at *p* < 0.05) are indicated by yellow color and arrows on axial MRI. (**A**) shows the correlations in the left medial temporal pole (arrows without *) and fusiform gyrus (an arrow with *) and (**B**) shows those in the left medial temporal pole (arrows). L and R indicate the left and right sides of the hemispheres, respectively. Each value below each brain slice indicates each value in MNI z-coordinates.

**Figure 3:**
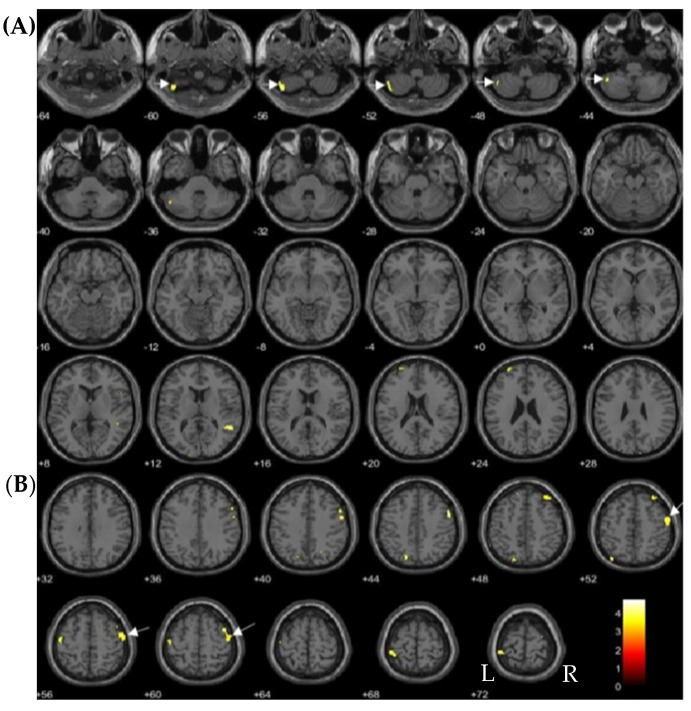
Brain regions positively correlated with 1,2,3,4,7,8-HexaCDD (FDR-corrected at *p* < 0.05) are indicated by yellow color and arrows on axial MRI. (**A**) shows the correlations in the left cerebellum lobule VII (arrows) and (**B**) shows those in the right middle frontal gyrus (arrows). L and R indicate the left and right sides of the hemispheres, respectively. Each value below each brain slice indicates each value in MNI z-coordinates.

Regions with low gray matter volume (inverse correlations) and high gray matter volume (positive correlations) in association with high dioxin exposure are shown in Table 3 and Figure 1, Figure 2 and Figure 3. Figure 1 shows an example of inverse correlation between the voxel values of the left fusiform gyrus at [−27 (x), 8 (y), −47 (z)] and blood TCDD levels (see below for the details).

Volume in the anterior temporal cortex including the left medial temporal pole and fusiform gyrus showed significant inverse correlations with TCDD (*p* < 0.05, FDR-corrected; Table 3, Figure 2A). The left medial temporal pole was significantly inversely correlated with TEQ-PCDDs (*p* < 0.05, FDR-corrected; Table 3, Figure 2B). In contrast, the left cerebellum and right middle frontal gyrus volume were significantly positively correlated with 1,2,3,4,7,8-HxCDD exposure (*p* < 0.05, FDR-corrected; Table 3, Figure 3).

### 3.3. Comparisons of Global and Regional Brain Volumes between Men with and without Possible Perinatal Dioxin Exposure

#### 3.3.1. Global Brain Volume Analyses

Total gray matter volume was significantly higher in men supposed to have been subject to perinatal dioxin exposure than in men without perinatal exposure (*p* = 0.005; effect size (ES) = 0.252; Table 4). However, no significant differences in white matter volume and total cerebrospinal fluid were observed between men with and without perinatal dioxin exposure. Total brain volume and total intracranial volume were significantly higher in men with perinatal exposure than in men without exposure (*p* = 0.020, ES = 0.178 for total brain volume; *p* = 0.034, ES = 0.151 for total intracranial volume; Table 4).

#### 3.3.2. VBM Analysis; Brain Regions in Which Gray Matter Volume Differed between Men with and without Estimated Perinatal Dioxin Exposure

**Figure 4:**
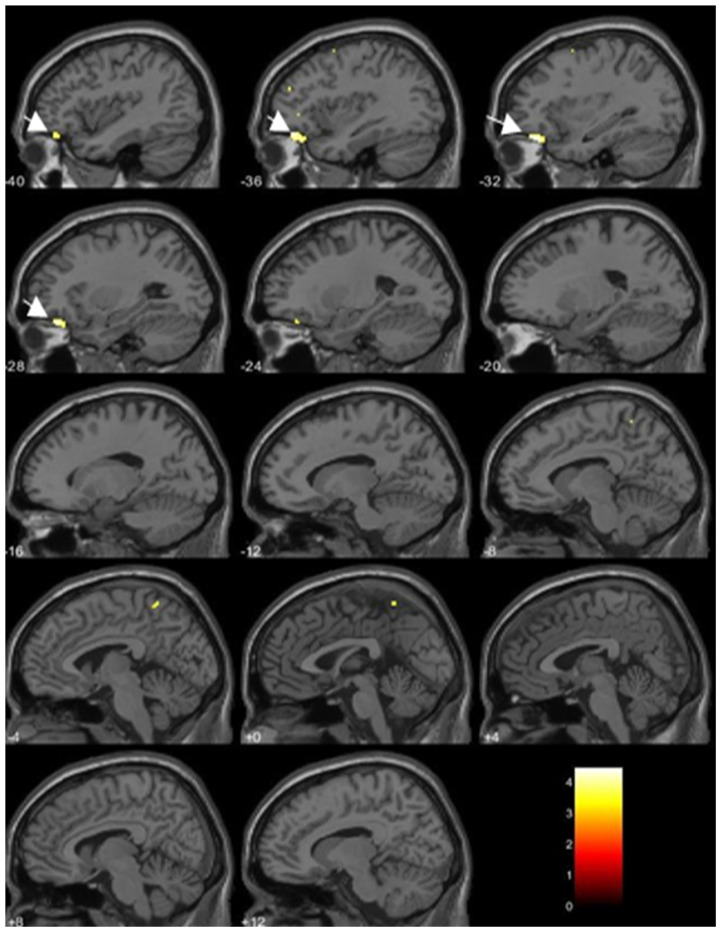
Brain regions associated with perinatal dioxin exposure (the left inferior frontal gyrus pars orbitalis indicated by yellow color and arrows on sagittal MRI slices). The volume was significantly lower in men with estimated perinatal dioxin exposure than in men without perinatal exposure (FDR-corrected at *p* < 0.05). Each value below each brain slice indicates each value in MNI x-coordinates.

The gray matter volume of the left inferior frontal gyrus (IFG) pars orbitalis was significantly lower in men with perinatal dioxin exposure than in with men without perinatal exposure after adjusting for total intracranial volume and age (*p* < 0.05, FDR-corrected; Table 5, Figure 4). However, no area showed a higher volume in men with perinatal exposure in comparison with men without exposure (Table 5).

## 4. Discussion

### 4.1. Relationships between Blood Dioxin Levels and Relevant Factors

In the present study, the geometric means of TCDD in the blood of the present subjects were 2.5-fold and 4.3-fold higher than those in the Phu Cat hotspot of dioxin contamination and a non-polluted area in Vietnam, respectively [20]. In particular, the blood TCDD levels were significantly higher in soldiers and mechanics who worked on machines belonging to the airbase than in those with other occupations. Similarly, Van Manh et al. (2021) reported TCDD concentrations in the blood of military workers in three Vietnamese airbases exposed to dioxins and found the highest concentrations in those who worked in Bien Hoa airbase [4]. These results suggest that a job related to an airbase is an important relevant factor for high levels of TCDD in men.

Consumption of foods such as vegetables and fish grown within the Bien Hoa airbase was also a significant factor relevant to high TCDD levels. Although the growing and catching of vegetables and fish within the airbase is forbidden, some people still consume vegetables and fish grown within it, thereby increasing the dioxin burden on their body. In addition, the use of herbicides and pesticides for agriculture in and around Bien Hoa airbase could be also associated with high levels of TCDD in the blood of the present subjects. However, agricultural chemicals produced in recent years should not be contaminated with TCDD, and should not therefore be a source of TCDD exposure. The enhanced levels in those working with herbicides and pesticides may be a factor co-incident with working with TCDD-contaminated soil in their farming areas, possibly from exposure to dust containing contaminated soil particles. These results suggest that TCDD originating from Agent Orange still remains a source of exposure to dioxin contamination in Bien Hoa airbase, and that TCDD in blood may reflect exposure levels in adulthood.

### 4.2. Regional Brain Volume Changes Associated with Blood Dioxin Levels

We showed that high TCDD and TEQ-PCDD levels in the blood were significantly associated with low gray matter volume in the left medial temporal pole and fusiform gyrus. Conversely, high 1,2,3,4,7,8-HxCDD exposure was associated with gray matter volume in the left cerebellum lobule VII and the right middle frontal gyrus. However, no significant correlations between blood dioxin levels and global volumes were found. These results suggest that dioxin exposure in adulthood might affect regional brain structure to alter brain volumes.

Previously, a follow-up study performed on workers in the Czech Republic 35 years after exposure to high levels of TCDD reported alteration of EEG and visual evoked potentials, and reduction of perfusion in various locations in the brain [21]. However, the effects of dioxin exposure on alterations to regional brain volumes are unknown. To our knowledge, this is the first study reporting morphological brain alterations associated with dioxin exposure. We found that opposing effects were associated with high dioxin levels, observing both volume increases and volume decreases. Previous animal studies suggested that the effects of persistent organic pollutants (POPs), including dioxins, might be non-monotonic: POPs exert both promoting and suppressing effects on neuronal dendritic growth, depending on their dose and the brain region, which may lead to brain volume increases and decreases, respectively [22,23,24].

We found that the gray matter volume in the fusiform gyrus was significantly inversely correlated with blood TCDD level. The fusiform gyrus is a large region in the temporal cortex implicated in high-level visual functions such as face perception, object recognition, and reading [25]. Grecucci et al. (2016) reported that some brain regions such as the middle frontal gyrus, fusiform gyrus, and cerebellum exhibited functional and structural abnormalities in patients with ASD in comparison with controls, referring to these regions as an autism-specific structural network [26]. The cerebellum is active in cognitive functions (including language) and executive functions [27], which might be disturbed in ASD. Another finding of our study is the association between low gray matter volume in the medial temporal pole and high exposure to TCDD and TEQ-PCDDs. The medial temporal pole may be involved in olfactory processing and connects with the orbitofrontal cortex and other emotion-related areas [28]. These results indicated that dioxin exposure in adulthood, including exposure to TCDD and 1,2,3,4,7,8-HxCDD, induced brain structural alterations in the areas involved in cognitive and emotional functions. Further following-up studies of our subjects with an assessment of cognitive and emotional functions are required to determine if these structural changes induce cognitive and emotional impairments.

### 4.3. Global and Regional Brain Volume Alterations Associated with Estimated Perinatal Dioxin Exposure

Our results indicate that perinatal dioxin exposure, which was estimated on the basis of the mother’s residency in Bien Hoa during pregnancy, was significantly associated with increased global gray matter volume, total brain volume, and total intracranial volume. To our knowledge, no previous study has investigated the effects of perinatal dioxin exposure on structural brain changes in humans. Although some previous animal studies reported reduced cortical thickness in several brain areas and changes in cortical cell numbers and cell distributions in TCDD-exposed rats [29,30], recent animal studies suggest that dioxins in low doses may lead to neuronal dendritic overgrowth [22,24], which may in turn lead to increases in brain volume.

Our previous study found that perinatal TCDD exposure increased autistic traits in Vietnamese children [5]. It was also reported that Vietnamese children perinatally exposed to dioxin showed disturbed mirror neuron activity [31], which is one of the social cognitive deficits present in autism. Moreover, extensive studies have reported brain enlargement in children [32,33,34], adolescents, and adults with autism [35,36]. In a VBM study of 833 children and adults, Riddle et al. (2017) recognized higher total brain volume and gray matter volume of approximately 1–2% in the ASD group compared with the typically-developing group [11]. Consistently, an animal model of autism showed dendritic overgrowth [37]. These similar results (increased brain volume) suggest that perinatal dioxin exposure and autism may share a similar pathogenesis: dendritic overgrowth could be a cause of pathogenesis resulting in brain enlargement and cognitive deficits.

Additionally, to identify regional gray matter abnormalities in men subjected to perinatal dioxin exposure, we applied VBM analysis. After adjusting for total intracranial volume and age, this analysis showed regional gray matter volume reduction in the left IFG pars orbitalis compared with a group without such exposure. The IFG pars orbitalis is important in the comprehension of emotional signals and semantic communication in humans [38]. Salmond et al. (2003) reported abnormality of orbitofrontal cortical volume in adolescents with ASD [39]. Furthermore, we also reported that dioxin exposure was associated with attention deficit hyperactivity disorder (ADHD) traits in Vietnamese children [40]. Several studies in ADHD children also demonstrated reduced gray matter volume in the left orbitofrontal cortex relative to healthy children [41,42]. Volume reduction of the left IFG pars orbitalis may be associated with symptoms of inattention and/or impulsivity in ADHD [42]. Taking these findings together, perinatal dioxin exposure may increase total gray matter volume as well as total brain volume, and may also decrease gray matter volume in the IFG pars orbitalis, suggesting that perinatal dioxin exposure affects cognitive and social functions by altering global and local brain development, leading to adverse neurodevelopment and traits, such as ASD and ADHD.

### 4.4. Limitations

One limitation of our study is the use of the residency in Bien Hoa of the subjects’ mothers to estimate perinatal dioxin exposure. A previous study reported high levels of TCDD in breast milk in Bien Hoa residents (see Materials and Methods, Section 2.1), suggesting that our subjects were exposed to dioxins during the perinatal period. Furthermore, our previous studies reported that dioxin levels in breast milk were associated with neurodevelopment of children [5,6,7,8]. The present results indicated that the residency in Bien Hoa of the subjects’ mothers, which suggests that the subjects were subjected to perinatal exposure to dioxins, was significantly associated with changes in gray matter volumes that were not associated with the adulthood blood dioxin levels of the subjects. These findings suggest possible effects of perinatal exposure to dioxins on brain morphometry although dose-effect relationships are unknown. We previously reported adverse effects of perinatal dioxin exposure on the neurodevelopment of Vietnamese children. However, MRI examination of children is difficult in Vietnam because of the requirement for sedation. In the future, we will perform MRI studies in these subjects when they become adolescents, to investigate the presence of brain structural alterations associated with perinatal dioxin exposure. The small sample size is also another limitation of the present study.

We also presented regional structural alterations in the brain associated with blood dioxin levels that were different from those associated with perinatal exposure, suggesting the neurotoxic effects of dioxin on the developed brain in adulthood. However, we did not examine the subjects’ cognitive functions and emotional behavior in the present study. In future, we will investigate psychiatric symptoms and behavior in residents of Bien Hoa to clarify the effects of dioxins on the mental health of adults without perinatal dioxin exposure.

## 5. Conclusions

The present results demonstrated that the residency in Bien Hoa of the subjects’ mothers was significantly associated with changes in gray matter volumes (i.e., increased global gray matter volume as well as decreased gray matter volume in the left IFG pars orbitalis), which was not associated with the present blood dioxin levels of the subjects. This finding suggests the possible effects of perinatal exposure to dioxins on gray matter volumes. Dioxin exposure in adulthood, indicated by blood levels, was associated with low gray matter volume in the fusiform gyrus and the medial temporal pole, but high gray matter volume in the middle frontal gyrus and cerebellum. These results suggest that dioxin exposure during the perinatal period and in adulthood is associated with regional brain alterations, which might lead to cognitive deficits and unusual social emotional behavior (increased autistic traits) in Vietnamese men living in a hot spot of dioxin contamination.

## Figures and Tables

**Table 1:** Dioxin and non-ortho-PCB levels in blood (*n* = 32).

Dioxin Congeners	LOD (ppt)	Below LOD		GM	GSD	Min	Max
PCDD Congeners (pg/g Lipid)		No.	%				
2,3,7,8-TCDD	0.03	2	6.3	6.4	2.1	1.5	56.2
1,2,3,7,8-PeCDD	0.03	0	0	10.6	1.5	4.4	22.4
1,2,3,4,7,8-HxCDD	0.03	2	6.3	6.1	1.4	3.0	11.7
1,2,3,6,7,8-HxCDD	0.02	0	0	13.3	1.5	5.5	37.2
1,2,3,7,8,9-HxCDD	0.03	0	0	7.2	1.6	3.1	31.6
1,2,3,4,6,7,8-HpCDD	0.04	0	0	36.2	1.8	12.0	295
OctaCDD	0.04	0	0	1027	1.6	490	2570
PCDF congeners (pg/g lipid)							
2,3,7,8-TCDF	0.02	2	6.3	4.4	1.6	1.6	8.3
1,2,3,7,8-PeCDF	0.03	7	21.9	4.5	1.7	1.3	10.2
2,3,4,7,8-PeCDF	0.04	0	0	13.6	1.3	6.6	21.9
1,2,3,4,7,8-HxCDF	0.02	0	0	14.9	1.4	7.2	30.2
1,2,3,6,7,8-HxCDF	0.02	0	0	10.8	1.5	2.9	19.1
1,2,3,7,8,9-HxCDF	0.02	13	40.6	3.8	2.0	0.9	67.6
2,3,4,6,7,8-HxCDF	0.02	1	3.1	3.8	1.6	1.3	9.8
1,2,3,4,6,7,8-HpCDF	0.02	0	0	13.6	1.6	5.2	50.1
1,2,3,4,7,8,9-HpCDF	0.04	10	31.3	4.8	1.8	1.1	15.1
OctaCDF	0.07	15	46.9	10.0	1.7	2.2	21.4
Nonortho-PCB (pg/g lipid)							
TCB #77	0.05	0	0	56.8	1.6	17.8	186
TCB #81	0.05	31	96.9	ND	ND	ND	ND
PeCB #126	0.28	30	93.8	ND	ND	ND	ND
HxCB #169	0.05	2	6.3	43.6	1.7	16.2	145
TEQs (pg-TEQ/g lipid)							
TEQ-PCDDs				21.8	1.5	9.1	69.2
TEQ-PCDFs				8.6	1.3	5.0	14.1
TEQ-PCDDs/Fs				30.8	1.4	14.5	74.1
TEQ-nonortho PCBs				1.1	3.8	0.01	5.89
TEQ-PCDDs/Fs/nonorthoPCBs				32.4	1.4	14.5	79.4

*n*: number of subjects; GM: geometrical mean; GSD: geometrical standard deviation; Min: minimum; Max: maximum. LOD (ppt): limit of detection (ppt: parts per trillion): it was defined as a signal-to-noise (S/N) ratio of peak height of chromato-gram = 3. Average of LODs in measurements of each dioxin congener is shown. ND: not detected.

**Table 2:** Characteristics of the subjects (*n* = 32).

Characteristics	Mean, [*n*]	SD, (%)	Min	Max
Age (years)	35.5	5.9	25.3	49
Education (years)	11.9	3.1	1	16
Smoking	[15]	(46.9)		
Alcohol consumption	[26]	(81.3)		
Weight (kilogram)	66.3	9.4	46	81.2
Height (cm)	165.5	5.0	154	178
BMI	24.2	3.0	17.1	28.2
Right hand dominant	[28]	(87.5)		
Length of residency (years)	22.1	14.6	1	44
Used herbicides	[10]	(31.3)		
Worked nearby industrial park	[15]	(46.9)		
Job (% of jobs related to the airbase)	[5]	(15.6)		
Their mothers lived in Bien Hoa during pregnancy	[12]	(37.5)		

*n*: number of subjects; SD: standard deviation; BMI: body mass index.

**Table 3:** Brain regions significantly correlated with blood levels of dioxin congeners in men after adjusting for total intracranial volume and age (FDR-corrected at *p* < 0.05).

Dioxin Congeners	Brain Regions	No of Voxels in Each Cluster (k)	Peak Z Scores	MNI Coordinates		
				x	y	z
Inverse correlations						
TCDD	Anterior temporal cortex	905				
	(Left medial temporal pole)		3.81	−41	20	−38
	(Left fusiform gyrus)		3.90	−27	8	−47
TEQ-PCDDs	Left medial temporal pole	333	3.63	−39	21	−38
Positive correlations						
1,2,3,4,7,8-HxCDD	Left cerebellum lobule VII	373	3.87	−42	−60	−57
	Right middle frontal gyrus	505	3.86	41	6	60

FDR = false discovery rate; MNI = Montreal Neurological Institute.

**Table 4:** Adjusted comparisons of global brain volumes between men with and without perinatal dioxin exposure estimated according to their mothers’ residency in Bien Hoa during pregnancy.

Perinatal Dioxin Exposure	Without *(n* = 20)			With *(n* = 12)			*p*-Value	ES
Global Volumes	Adj Mean	95%CI		Adj Mean	95%CI			
		Lower	Upper		Lower	Upper		
Gray matter (GM) (cm^3^)	615	601	629	651	632	670	0.005	0.252
White matter (WM) (cm^3^)	527	508	545	551	527	576	0.118	0.085
Cerebrospinal fluid (CSF) (cm^3^)	300	283	317	306	284	329	0.651	0.007
Total brain volume (TBV) (cm^3^)	1142	1111	1172	1202	1163	1242	0.020	0.178
Total intracranial volume (TIV) (cm^3^)	1443	1401	1484	1517	1463	1570	0.034	0.151

*n*: number of subjects; adj mean: adjusted mean; CI: confidence interval; ES: effect size. Covariates: age (years) and height (cm).

**Table 5:** Brain regions showing significant contrasts in volume between men with and without perinatal dioxin exposure after adjusting for total intracranial volume and age (FDR-corrected at *p* < 0.05).

Brain Regions	No of Voxels in Each Cluster (k)	Peak Z Scores	MNI Coordinates		
			x	y	z
Without exposure > With exposure					
Left inferior frontal gyrus pars orbitalis	414	3.86	−32	39	−23
With exposure > Without exposure					
No brain region	-	-	-	-	-

FDR = false discovery rate; MNI = Montreal Neurological Institute. Perinatal exposure was estimated on their mothers’ residency in Bien Hoa during pregnancy. “-“ indicates that no significant correlations were detected.

## Data Availability

The data presented in this study are available on request to the corresponding author. The data are not publicly available due to the personal information (MRI data).

## References

[1] Dwernychuk L.W. (2005). Dioxin hot spots in Vietnam. Chemosphere.

[2] The Office of the Vietnam National Steering Committee 33, Hatfield Consultants (2011). Environmental and Human Health Assessment of Dioxin Contamination at Bien Hoa Airbase, Viet Nam.

[3] Van Luong H., Tai P.T., Nishijo M., Trung D.M., Thao P.N., Van Son P., Van Long N., Linh N.T., Nishijo H. (2018). Association of dioxin exposure and reproductive hormone levels in men living near the Bien Hoa airbase, Vietnam. Sci. Total Environ..

[4] Van Manh P., Tai P.T., Phuong N.M., Nishijo M., Trung D.M., Thao P.N., Son H.A., Van Tuan T., Van Chuyen N., Van Long N. (2021). Serum dioxin concentrations in military workers at three dioxin-contaminated airbases in Vietnam. Chemosphere.

[5] Nishijo M., Pham T.T., Nguyen A.T., Tran N.N., Nakagawa H., Hoang L.V., Tran A.H., Morikawa Y., Ho M.D., Kido T. (2014). 2,3,7,8-Tetrachlorodibenzo-p-dioxin in breast milk increases autistic traits of 3-year-old children in Vietnam. Mol. Psychiatry.

[6] Pham N.T., Nishijo M., Pham T.T., Tran N.N., Le V.Q., Tran H.A., Phan H.A.V., Nishino Y., Nishijo H. (2019). Perinatal dioxin exposure and neurodevelopment of 2-year-old Vietnamese children in the most contaminated area from Agent Orange in Vietnam. Sci. Total Environ..

[7] Nghiem G.T., Nishijo M., Pham T.N., Ito M., Pham T.T., Tran A.H., Nishimaru H., Nishino Y., Nishijo H. (2019). Adverse effects of maternal dioxin exposure on fetal brain development before birth assessed by neonatal electroencephalography (EEG) leading to poor neurodevelopment; a 2-year follow-up study. Sci. Total Environ..

[8] Pham N.T., Nishijo M., Nghiem T.T.G., Pham T.T., Tran N.N., Le V.Q., Vu T.H., Tran H.A., Phan H.A.V., Do Q. (2021). Effects of perinatal dioxin exposure on neonatal electroencephalography (EEG) activity of the quiet sleep stage in the most contaminated area from Agent Orange in Vietnam. Int. J. Hyg. Environ. Health.

[9] Albajara Saenz A., Van Schuerbeek P., Baijot S., Septier M., Deconinck N., Defresne P., Delvenne V., Passeri G., Raeymaekers H., Slama H. (2020). Disorder-specific brain volumetric abnormalities in Attention-Deficit/Hyperactivity Disorder relative to Autism Spectrum Disorder. PLoS ONE.

[10] Lim L., Chantiluke K., Cubillo A.I., Smith A.B., Simmons A., Mehta M.A., Rubia K. (2015). Disorder-specific grey matter deficits in attention deficit hyperactivity disorder relative to autism spectrum disorder. Psychol. Med..

[11] Riddle K., Cascio C.J., Woodward N.D. (2017). Brain structure in autism: A voxel-based morphometry analysis of the Autism Brain Imaging Database Exchange (ABIDE). Brain Imaging Behav..

[12] Raznahan A., Greenstein D., Lee N.R., Clasen L.S., Giedd J.N. (2012). Prenatal growth in humans and postnatal brain maturation into late adolescence. Proc. Natl. Acad. Sci. USA.

[13] Schecter A., Dai L.C., Papke O., Prange J., Constable J.D., Matsuda M., Thao V.D., Piskac A.L. (2001). Recent dioxin contamination from Agent Orange in residents of a southern Vietnam city. J. Occup. Environ. Med..

[14] Van den Berg M., Birnbaum L.S., Denison M., De Vito M., Farland W., Feeley M., Fiedler H., Hakansson H., Hanberg A., Haws L. (2006). The 2005 World Health Organization Reevaluation of Human and Mammalian Toxic Equivalency Factors for Dioxins and Dioxin-Like Compounds. Toxicol. Sci..

[15] Ashburner J., Friston K.J. (2005). Unified segmentation. NeuroImage.

[16] Ashburner J. (2007). A fast diffeomorphic image registration algorithm. NeuroImage.

[17] Nordenskjold R., Malmberg F., Larsson E.M., Simmons A., Brooks S.J., Lind L., Ahlstrom H., Johansson L., Kullberg J. (2013). Intracranial volume estimated with commonly used methods could introduce bias in studies including brain volume measurements. NeuroImage.

[18] Genovese C.R., Lazar N.A., Nichols T. (2002). Thresholding of statistical maps in functional neuroimaging using the false discovery rate. NeuroImage.

[19] Eickhoff S.B., Stephan K.E., Mohlberg H., Grefkes C., Fink G.R., Amunts K., Zilles K. (2005). A new SPM toolbox for combining probabilistic cytoarchitectonic maps and functional imaging data. NeuroImage.

[20] Manh H.D., Kido T., Okamoto R., Xianliang S., Anhle T., Supratman S., Maruzeni S., Nishijo M., Nakagawa H., Honma S. (2014). Serum dioxin levels in Vietnamese men more than 40 years after herbicide spraying. Environ. Sci. Technol..

[21] Urban P., Pelclova D., Lukas E., Kupka K., Preiss J., Fenclova Z., Smerhovsky Z. (2007). Neurological and neurophysiological examinations on workers with chronic poisoning by 2,3,7,8-TCDD: Follow-up 35 years after exposure. Eur. J. Neurol..

[22] Kimura E., Kubo K., Matsuyoshi C., Benner S., Hosokawa M., Endo T., Ling W., Kohda M., Yokoyama K., Nakajima K. (2015). Developmental origin of abnormal dendritic growth in the mouse brain induced by in utero disruption of aryl hydrocarbon receptor signaling. Neurotoxicol. Teratol..

[23] Gileadi T.E., Swamy A.K., Hore Z., Horswell S., Ellegood J., Mohan C., Mizuno K., Lundebye A.K., Giese K.P., Stockinger B. (2021). Effects of Low-Dose Gestational TCDD Exposure on Behavior and on Hippocampal Neuron Morphology and Gene Expression in Mice. Environ. Health Perspect..

[24] Latchney S.E., Majewska A.K. (2021). Persistent organic pollutants at the synapse: Shared phenotypes and converging mechanisms of developmental neurotoxicity. Dev. Neurobiol..

[25] Weiner K.S., Zilles K. (2016). The anatomical and functional specialization of the fusiform gyrus. Neuropsychologia.

[26] Grecucci A., Rubicondo D., Siugzdaite R., Surian L., Job R. (2016). Uncovering the Social Deficits in the Autistic Brain. A Source-Based Morphometric Study. Front. Neurosci..

[27] Becker E.B., Stoodley C.J. (2013). Autism spectrum disorder and the cerebellum. Int. Rev. Neurobiol..

[28] Fan L., Wang J., Zhang Y., Han W., Yu C., Jiang T. (2014). Connectivity-based parcellation of the human temporal pole using diffusion tensor imaging. Cereb. Cortex.

[29] Zareba G., Hojo R., Zareba K.M., Watanabe C., Markowski V.P., Baggs R.B., Weiss B. (2002). Sexually dimorphic alterations of brain cortical dominance in rats prenatally exposed to TCDD. J. Appl. Toxicol..

[30] Hojo R., Zareba G., Kai J.W., Baggs R.B., Weiss B. (2006). Sex-specific alterations of cerebral cortical cell size in rats exposed prenatally to dioxin. J. Appl. Toxicol..

[31] Vu H.T., Nishijo M., Pham T.N., Pham-The T., Hoanh L.V., Tran A.H., Tran N.N., Nishino Y., Do Q., Nishijo H. (2021). Effects of perinatal dioxin exposure on mirror neuron activity in 9-year-old children living in a hot spot of dioxin contamination in Vietnam. Neuropsychologia.

[32] Sparks B.F., Friedman S.D., Shaw D.W., Aylward E.H., Echelard D., Artru A.A., Maravilla K.R., Giedd J.N., Munson J., Dawson G. (2002). Brain structural abnormalities in young children with autism spectrum disorder. Neurology.

[33] Palmen S.J., Hulshoff Pol H.E., Kemner C., Schnack H.G., Durston S., Lahuis B.E., Kahn R.S., Van Engeland H. (2005). Increased gray-matter volume in medication-naive high-functioning children with autism spectrum disorder. Psychol. Med..

[34] Stanfield A.C., McIntosh A.M., Spencer M.D., Philip R., Gaur S., Lawrie S.M. (2008). Towards a neuroanatomy of autism: A systematic review and meta-analysis of structural magnetic resonance imaging studies. Eur. Psychiatry.

[35] Freitag C.M., Luders E., Hulst H.E., Narr K.L., Thompson P.M., Toga A.W., Krick C., Konrad C. (2009). Total brain volume and corpus callosum size in medication-naive adolescents and young adults with autism spectrum disorder. Biol. Psychiatry.

[36] Hazlett H.C., Poe M.D., Gerig G., Smith R.G., Piven J. (2006). Cortical gray and white brain tissue volume in adolescents and adults with autism. Biol. Psychiatry.

[37] Cheng N., Alshammari F., Hughes E., Khanbabaei M., Rho J.M. (2017). Dendritic overgrowth and elevated ERK signaling during neonatal development in a mouse model of autism. PLoS ONE.

[38] Belyk M., Brown S., Lim J., Kotz S.A. (2017). Convergence of semantics and emotional expression within the IFG pars orbitalis. NeuroImage.

[39] Salmond C.H., de Haan M., Friston K.J., Gadian D.G., Vargha-Khadem F. (2003). Investigating individual differences in brain abnormalities in autism. Philos. Trans. R. Soc. Lond. B Biol. Sci..

[40] Pham-The T., Nishijo M., Pham-Ngoc T., Vu-Thi H., Tran-Ngoc N., Tran-Hai A., Hoang-Van L., Nishino Y., Nishijo H. Effects of prenatal dioxin exposure on children behaviors at 8 years of age of age. Proceedings of the 39th International Symposium on Halogenated Persistent Organic Pollutants—Dioxin in 2019.

[41] Kumar U., Arya A., Agarwal V. (2017). Neural alterations in ADHD children as indicated by voxel-based cortical thickness and morphometry analysis. Brain Dev..

[42] Nickel K., Tebartz van Elst L., Manko J., Unterrainer J., Rauh R., Klein C., Endres D., Kaller C.P., Mader I., Riedel A. (2018). Inferior Frontal Gyrus Volume Loss Distinguishes Between Autism and (Comorbid) Attention-Deficit/Hyperactivity Disorder—A FreeSurfer Analysis in Children. Front. Psychiatry.

